# A Serum Metabolomics Classifier Derived from Elderly Patients with Metastatic Colorectal Cancer Predicts Relapse in the Adjuvant Setting

**DOI:** 10.3390/cancers13112762

**Published:** 2021-06-02

**Authors:** Samantha Di Donato, Alessia Vignoli, Chiara Biagioni, Luca Malorni, Elena Mori, Leonardo Tenori, Vanessa Calamai, Annamaria Parnofiello, Giulia Di Pierro, Ilenia Migliaccio, Stefano Cantafio, Maddalena Baraghini, Giuseppe Mottino, Dimitri Becheri, Francesca Del Monte, Elisangela Miceli, Amelia McCartney, Angelo Di Leo, Claudio Luchinat, Laura Biganzoli

**Affiliations:** 1 Department of Medical Oncology, New Hospital of Prato S. Stefano, 59100 Prato, Italy; luca.malorni@uslcentro.toscana.it (L.M.); elena2.mori@uslcentro.toscana.it (E.M.); vanessa.calamai@uslcentro.toscana.it (V.C.); annamaria.parnofiello@uslcentro.toscana.it (A.P.); giulia.dipierro@uslcentro.toscana.it (G.D.P.); francesca.delmonte@uslcentro.toscana.it (F.D.M.); elisangela.miceli@uslcentro.toscana.it (E.M.); amelia.mccartney@monashhealth.org (A.M.); angelo.dileo@uslcentro.toscana.it (A.D.L.); laura.biganzoli@uslcentro.toscana.it (L.B.); 2 Magnetic Resonance Center, University of Florence, 50019 Sesto Fiorentino, Italy; vignoli@cerm.unifi.it (A.V.); tenori@cerm.unifi.it (L.T.); luchinat@cerm.unifi.it (C.L.); 3 Department of Chemistry “Ugo Schiff”, University of Florence, 50019 Sesto Fiorentino, Italy; 4 Bioinformatics Unit, Medical Oncology Department, New Hospital of Prato S. Stefano, 59100 Prato, Italy; chiara.biagioni@uslcentro.toscana.it; 5 “Sandro Pitigliani” Translational Research Unit, New Hospital of Prato, Stefano, 59100 Prato, Italy; ilenia.migliaccio@uslcentro.toscana.it; 6 Department of Medicine (DAME), University of Udine, 33100 Udine, Italy; 7 Department of Surgery, New Hospital of Prato S. Stefano, 59100 Prato, Italy; stefano.cantafio@uslcentro.toscana.it (S.C.); maddalena.baraghini@uslcentro.toscana.it (M.B.); 8 Department of Geriatrics, New Hospital of Prato S. Stefano, 59100 Prato, Italy; giuseppe.mottino@uslcentro.toscana.it (G.M.); dimitri.becheri@uslcentro.toscana.it (D.B.); 9 School of Clinical Sciences, Monash University, 3168 Clayton, Australia; 10 Consorzio Interuniversitario Risonanze Magnetiche di Metallo Proteine (C.I.R.M.M.P.), 50019 Sesto Fiorentino, Italy

**Keywords:** metabolomics, colorectal cancer, recurrence, prognosis, NMR spectroscopy, elderly

## Abstract

**Simple Summary:**

Around 30–40% of patients with early stage colorectal cancer (eCRC) experience relapse after surgery. Current recommendations for adjuvant therapy are based on suboptimal risk-stratification tools. In elderly patients, risk of relapse assessment is particularly important to ultimately avoid unnecessary chemotherapy-related toxicity in this frailer population. Serum metabolomics via NMR spectroscopy may improve risk stratification by identifying patients with residual micrometastases after surgery and thus at higher risk of relapse. We evaluated the serum metabolomic fingerprints of 94 elderly patients with eCRC (65 relapse free and 29 relapsed), and of 75 elderly patients with metastatic disease. Metabolomics efficiently discriminated patients with relapse-free eCRC from those with metastatic disease, correctly predicting relapse in 69% of relapsed eCRC patients. The metabolomic score was strongly and independently associated with prognosis. Our data suggest metabolomics as a valid addition to standard tools to refine risk stratification for eCRC and warrant further investigation.

**Abstract:**

Adjuvant treatment for patients with early stage colorectal cancer (eCRC) is currently based on suboptimal risk stratification, especially for elderly patients. Metabolomics may improve the identification of patients with residual micrometastases after surgery. In this retrospective study, we hypothesized that metabolomic fingerprinting could improve risk stratification in patients with eCRC. Serum samples obtained after surgery from 94 elderly patients with eCRC (65 relapse free and 29 relapsed, after 5-years median follow up), and from 75 elderly patients with metastatic colorectal cancer (mCRC) obtained before a new line of chemotherapy, were retrospectively analyzed via proton nuclear magnetic resonance spectroscopy. The prognostic role of metabolomics in patients with eCRC was assessed using Kaplan–Meier curves. PCA-CA-kNN could discriminate the metabolomic fingerprint of patients with relapse-free eCRC and mCRC (70.0% accuracy using NOESY spectra). This model was used to classify the samples of patients with relapsed eCRC: 69% of eCRC patients with relapse were predicted as metastatic. The metabolomic classification was strongly associated with prognosis (*p*-value 0.0005, HR 3.64), independently of tumor stage. In conclusion, metabolomics could be an innovative tool to refine risk stratification in elderly patients with eCRC. Based on these results, a prospective trial aimed at improving risk stratification by metabolomic fingerprinting (LIBIMET) is ongoing.

## 1. Introduction

Colorectal cancer (CRC) is the second most common cancer in developed countries, with approximately two-thirds of patients having resectable primary disease at diagnosis. Unfortunately, 30–40% of these patients will eventually experience relapse after surgery [1]. CRC is a heterogeneous entity that presents with different characteristics of clinical onset and individual treatment response, even at the same pathological stage. The assessment of recurrence risk in colon cancer is based primarily on pathological stage as defined by the TNM system based on the depth of tumor wall invasion, lymph node involvement, and distant metastasis [2,3]. This staging system is clinically useful and is highly associated with 5-year overall survival (OS), ranging from 92% in stage I disease to 11% at stage IV. Patients with stage II and III CRC comprise a largely heterogeneous group, with 5-year OS ranging between 50% and 90% [4]. 

The choice of adjuvant treatment is based on risk stratification; however, it is known that only a small proportion of patients benefit from adjuvant therapy, with the majority being already cured by primary surgery, and others experiencing later disease relapse despite having received adjuvant chemotherapy. Several data from randomized clinical studies have identified clear survival benefits of adjuvant chemotherapy in patients with stage III colon cancer [5,6,7,8,9,10]. Contrastingly, in stage II disease, the role of adjuvant chemotherapy is still controversial: the reported five-year survival ranges from 75% to 87.5%, with only 3.5–5% of patients benefiting from adjuvant chemotherapy [11]. In patients with clinicopathologically high-risk stage II disease (defined by T4 lesions, clinical presentation with bowel obstruction or perforation, fewer than 12 lymph nodes examined, and poor differentiation histology), decision-making around adjuvant chemotherapy must be discussed and evaluated. A high level of baseline carcinoembryonic antigen (i.e., >5 ng/L), large vessel invasion, and the presence of perineural and extramural vascular invasion are also considered risk factors associated with recurrence [2,12]. In stage III colon cancers, 50–60% of patients are cured by surgery alone and an additional 20% with adjuvant chemotherapy, with a significant proportion of patients who will derive no benefit from adjuvant therapy [11].

CRC is more frequent in older patients, but only a minority of older patients with CRC are enrolled in a clinic trial [13]. Pooled analyses of major randomized trials of adjuvant chemotherapy as well as real-world evidence show that elderly patients with early CRC derive similar benefit from adjuvant chemotherapy as compared to younger patients in stage III, with conflicting results in stage II [14,15,16,17,18]. Older patients are generally undertreated when compared with younger patients based on chronological age. However, available evidence underlines that one-third of elderly patients discontinue adjuvant therapy, due to poor compliance and toxicity [19,20,21].

In this context, refining the stratification of risk for patients eligible for post-operative treatment is an essential issue, particularly for elderly patients.

Several significant efforts to improve risk stratification in colorectal cancer have been made in the past years. Molecular assessment of colon cancer has been refined in the past decade, taking into consideration mismatch repair (MMR) status, as well as BRAF and KRAS mutations, all markers with proven prognostic value in terms of disease-free survival (DFS) and OS [8,22]. Nevertheless, the application of these markers in clinical practice to guide adjuvant therapy remains limited, due to the lack of rigorous assessment in large patient cohorts and subsequent implementation and validation [23,24,25]. Some data have demonstrated that patients with stage II eCRC and concurrent microsatellite instability (MSI) have a more favorable prognosis, and adjuvant chemotherapy with fluorouracil (FU) as a single agent may indeed have a detrimental effect in these patients [26,27,28]. Recently, the Immunoscore developed by Galon et al. [29] has been validated prospectively in a large population of patients with stage III disease and MSI assessment, but its definitive impact in patients with stage II and III eCRC treated with chemotherapy requires further evaluation [30]. Better identification of patients who will most benefit from adjuvant chemotherapy is an important goal, especially in older patients who are more vulnerable to treatment-related toxicity.

The risk of recurrence of CRC is considered as a function of the presence of micro-metastatic dissemination of cancer cells at diagnosis. The detection of micro-metastatic residual disease after surgical resection is a key challenge in the current treatment paradigm of eCRC. Recently, the presence of tumor-derived circulating DNA (ctDNA) in the bloodstream after surgery has shown potential in identifying patients with minimal residual disease, thus at high risk of relapse, independent of pathological stage [31,32,33,34,35,36]. In particular, two cohort studies, aimed at determining the prognostic value of ctDNA in patients with resected stage II and III eCRC who had at least one tumor-specific DNA mutation, achieved compelling results and launched a new generation of trials based on post-surgery ctDNA status for the choice of adjuvant treatment [33,35].

Metabolomics is an -omics science that employs analysis of the ensemble of metabolites present in a biological specimen, the metabolome [37,38]. The metabolome represents the downstream molecular result of genomic, proteomic, transcriptomic, and many other exogenous factors, and can therefore be thought of as a reliable reflection of any disease status or phenotype [39]. In particular, cancer metabolomics of systemic biofluids is a molecular representation not only of the tumor phenotype, but considers both the effects that a cancer exerts upon an individual, and the individual upon the cancer (i.e., as a tumor micro-environment, inflammatory and immune responses) [40,41]. For these reasons, metabolomics constitutes a powerful platform for discovering novel cancer biomarkers, and for providing further insights into the host–tumor biochemistry.

Several groups have characterized metabolomic changes related to CRC [42,43,44,45,46,47]. Our group has already established a reproducible method of quantifying the individual metabolomic fingerprint via nuclear magnetic resonance (NMR) spectroscopy and has demonstrated its ability to accurately discriminate between advanced and early breast cancer [48]. Furthermore, we have previously demonstrated that this metabolomic fingerprinting methodology can be used to predict the risk of disease recurrence in early breast cancer [49,50,51].

Here, we propose an NMR-based metabolomics study aimed at defining a serum metabolomic fingerprint able to discriminate early (eCRC) and metastatic (mCRC) colorectal cancer in elderly patients. Moreover, we used this fingerprint to identify patients with eCRC patients who are at higher risk of disease recurrence, who may therefore be more likely to benefit from adjuvant chemotherapy.

## 2. Materials and Methods

This retrospective study was a collaborative project between the Magnetic Resonance Centre of the University of Florence (Sesto Fiorentino, Italy) and the Sandro Pitigliani Medical Oncology Department, Hospital of Prato (Prato, Italy). Trials included in these analyses received approval from the local institutional ethics committee and were conducted in accordance with GCP and the principles of the Declaration of Helsinki. Written informed consent was prospectively obtained from all patients participating in these trials.

### 2.1. Patient Selection 

Serum samples from 169 elderly patients with CRC were collected. Of these, 94 were from patients with eCRC and 75 with mCRC. 

Early colon cancer is defined as early stage, localized, resectable disease without distant metastases at time of diagnosis, while metastatic colorectal cancer is defined as advanced disease, not resectable, with distant metastases.

**Figure 1:**
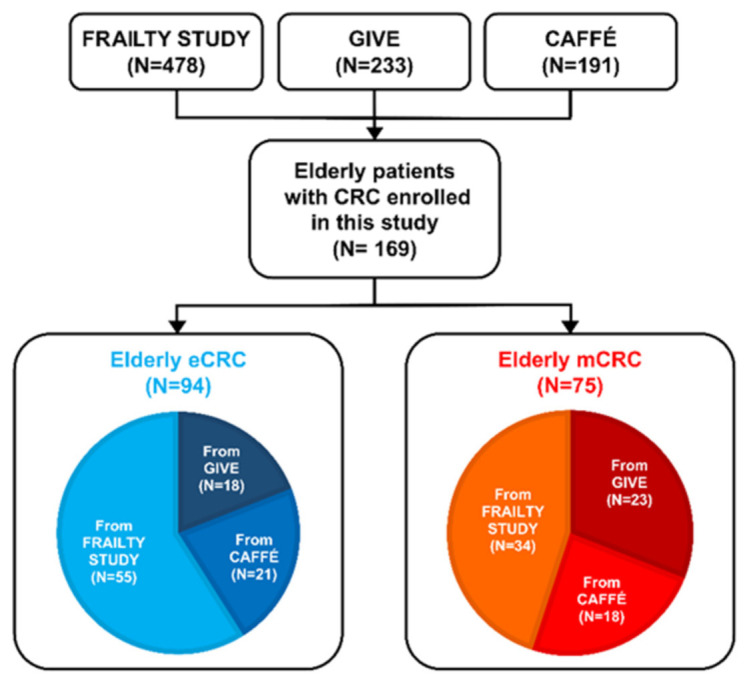
CRC patients enrolled in this study were divided in eCRC and mCRC according to the disease stage. Serum samples were pooled among three clinical trials (Frailty Study, GIVE, and CAFFÉ). The number of samples coming from each study is also reported.

There was a median follow up time of 5 years in the eCRC cohort, and 3.9 years in the mCRC cohort. Patients enrolled in this study were pooled from three clinical trials (Frailty study, GIVE and CAFFÈ trials) focused on elderly patients with solid malignancies (including colorectal cancer), in which serum samples for metabolomic analysis were prospectively collected; for the present analysis, only patients with colorectal cancer were selected (Figure 1). All patients were treated and followed at the Oncology Department of the Hospital of Prato between 2008 and 2018 (see patient distribution in Figure 1).

The first study prospectively evaluated the performance of three different geriatric tools in identifying frailty in elderly patient with early stage solid cancers [52]. The second study, “GIVE” (Impact of Geriatrician-implemented Interventions on chemotherapy delivery in Vulnerable Elderly patients with early or metastatic solid malignancies), is a multicentric randomized trial that aimed at evaluating the impact of geriatric guided interventions on chemotherapy delivery in patients aged 70 years or older who presented at least one deficit identified at a comprehensive geriatric assessment (CGA). Its parallel study (“CAFFE”: Chemotherapy delivery in Comprehensive Geriatric Assessment (CGA)-deFined Fit Elderly patients with early or metastatic solid tumors) evaluated chemotherapy compliance in fit patients aged 70 years or older.

Both CAFFE and GIVE targeted patients with early stage or advanced solid malignancies receiving chemotherapy in the neo/adjuvant setting, or as first- or second-line chemotherapy for advanced disease (NCT02785887). All patients included in the three trials were treated as per standard clinical practice.

All the patients included in the three studies were evalueted with comprehensive geriatric assessment (CGA) at study entry.

### 2.2. Serum Sample Collection and Storage

Serum samples were collected from all patients who consented to participate in each study. For each patient, one 10-mL overnight fasting peripheral blood sample was collected after surgery, prior to commencement of chemotherapy in early setting, and before chemotherapy commencement in the metastatic setting. Blood was centrifuged at room temperature for 10 min at 1500 g, then serum was collected, and 1-mL aliquots transferred into pre-labelled cryovials. Within one hour of collection, samples were frozen at −20 °C and then stored at −80 °C pending NMR analysis.

### 2.3. NMR Analysis

Serum samples were stored and prepared for NMR analysis according to the standard operating procedures [53]. One-dimensional 1H NMR spectra were acquired using a Bruker 600 MHz spectrometer (Bruker BioSpin, Rheinstetten, Germany) operating at 600.13 MHz proton Larmor frequency. Before measurement, for temperature equilibration at 310 K, samples were kept for at least five minutes inside the NMR probehead.

For each serum sample, three one-dimensional 1H NMR spectra with different pulse sequences were acquired, allowing the selective detection of different molecular components: a standard nuclear Overhauser effect spectroscopy pulse sequence NOESY 1Dpresat was applied to detect both signals of low (metabolites) and high (i.e., lipids, lipoproteins, proteins) molecular weight molecules. A standard spin echo Carr-Purcell-Meiboom-Gill 1D sequence (CPMG) and a standard diffusion-edited pulse sequence were used to selectively detect signals of low molecular weight metabolites and high molecular weight macromolecules, respectively.

An extended description of the sample preparation procedures, instrument configuration, and setting of the NMR parameters can be found in our previous publication [50].

### 2.4. NMR Spectra Processing

Free induction decays were multiplied by an exponential function equivalent to a 1.0 Hz line-broadening factor before applying Fourier transform. Transformed spectra were automatically corrected for phase and baseline distortions and calibrated (anomeric glucose ^1^H doublet at 5.24 ppm) using TopSpin 3.2 (Bruker Biospin, Rheinstetten, Germany). Each 1-D spectrum in the range between 0.2 and 10.0 ppm was segmented into 0.05 ppm chemical shift bins and the corresponding spectral areas were integrated using AssureNMR software (Bruker BioSpin, Rheinstetten, Germany). Spectral regions containing residual water signal and ethanol signals were removed and the dimension of the system was reduced to 156 bins. Probabilistic Quotient Normalization (PQN) [54] was applied on the data prior to pattern recognition.

### 2.5. Statistical Analysis

All data analysis was performed using the open source “R” statistical environment [55] (Version Microsoft R Open 3.5.1). Data reduction was obtained by means of the projection of the binning data matrix into the principal component analysis (PCA) subspace. Only the components that explain 99.9% of the variance were retained in the model and the canonical analysis (CA) was applied to obtain the supervised separation of the groups of interest. For the purpose of classification, we used the K-nearest neighbors (k-NN) [56] algorithm (k = 9) applied on the PCA-CA scores. The PCA-CA-kNN modelling was performed using an R script in-house developed. Sensitivity, specificity, and accuracy, calculated according to the standard definition for each model, were assessed by means of a leave-one-out cross-validation scheme (LOOCV, R script in-house developed).

Univariate analysis was conducted directly on the spectral regions associated to the metabolites present in all serum samples at concentrations above the detection limit (>1 μM). Metabolite signals were assigned on template one-dimensional NMR spectra by using the BIOREFCODE reference database (Bruker BioSpin, Rheinstetten, Germany) and the Human Serum Metabolome Database [57]. The spectral region related to 32 different metabolites was quantified by using a R script developed in-house. The Wilcoxon rank-sum test [58] (R package “stats”) was used to perform univariate analysis. False discovery rate correction (R package “stats”) was applied using the Benjamini–Hochberg method [59]: an adjusted *p*-value of 0.05 was deemed significant. Moreover, for each metabolite, effect size using Cliff’s delta was calculated by means of the R package “effsize”.

The recurrence-free interval (RFI) was defined as the time interval between the date of informed consent and colorectal cancer relapse. DFS was defined as the time from informed consent to disease progression or death. Cancer-specific survival (CSS) was defined as the time between informed consent and death after colorectal cancer relapse. Cases that remained colorectal cancer free were censored at the time of death or the last follow-up visit. OS was computed from the date of informed consent to death from any cause. The median follow-up time was estimated using the reverse Kaplan–Meier method censoring for death. The distributions of all outcomes were estimated using the Kaplan–Meier method and compared with the log-rank test (R packages “survival” and “survminer”).

Hazard ratios (HRs) and confidence intervals (CIs) were estimated using the Cox proportional hazards model. A multivariate Cox regression model was fitted to evaluate the independent effect of available covariates on RFI. Using stepwise selection, we created a model that includes all significant covariates, a *p*-value < 0.05 was used for both entry and stay criteria (R package “survival”). 

## 3. Results

### 3.1. Patient Characteristics

In total, 169 elderly patients aged ≥ 70 years with CRC prospectively enrolled in three onco-geriatric clinical trials were included in this study (Figure 1).

The median age at study entry was 78 years for patients with eCRC and 76 years in the mCRC cohort. Patients with eCRC were equally split between stage II (45%, *N* = 42) and III (43%, *N* = 41), with only three patients with stage I disease. In this cohort, the majority of patients had tumors localized in the left colon or rectum (61%, *N* = 57) and had pT3 disease (77%, *N* = 77), positive lymph node status (48%, *N* = 45), and grade 2 primary disease (74%, *N* = 67).

The major clinic-pathological characteristics of eCRC patients are reported in Table 1. Overall, 56 patients—25 with stage II and 31 with stage III disease—received an adjuvant fluoropyrimidine-based chemotherapy. Eight patients with rectal cancer received neoadjuvant chemo-radiotherapy.

At a median follow up of 5 years, 29 patients (31%) experienced disease relapse. Not unexpectedly, most of these patients had stage III disease (69%, *N* = 20), with recurrence-free interval (RFI) influenced by stage disease. None of the three patients with stage I disease recurred (Figure S1A). Similar results were observed in terms of disease-free survival (Figure S1B). Primary tumor location (left/rectum vs. right) did not have a significant impact on DFS and RFI (data not shown).

The metastatic cohort included 75 patients recruited before the start of systemic therapy (88%) or best supportive care (12%). The characteristics of the patients included the metastatic cohort are reported in Supplementary Materials Table S1. The metastatic cohort included 75 patients recruited before the start of systemic therapy (first-line: 77%) or best supportive care (12%). This cohort had an equal distribution of left-sided and right-sided primary site (*n* = 37 and 36, respectively). 

Overall, at a median follow up time of 3.9 years (2.8-NA 95% confidence interval), median PFS was 7.8 months in this population (95% confidence interval (CI) 6.4-10 months), with median OS of 14 months (95% CI 12-21 months) (Table S1).

### 3.2. Metabolomics Discrimination between Patients with Non-Relapsed eCRC and mCRC

**Figure 2:**
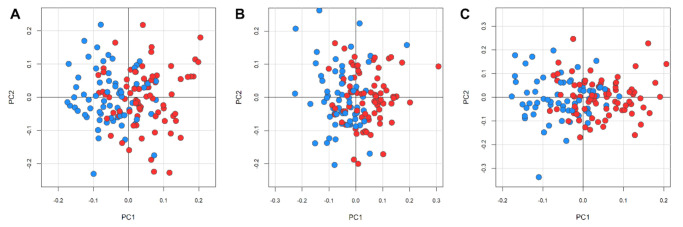
Clusterization of serum metabolomic fingerprints. Discrimination between patients free from eCRC disease relapse (light blue, dots, *n* = 65) and patients with mCRC (red dots, *n* = 75) using the PCA-CA-kNN analysis. Score plot of the first two principal components (**A**) NOESY1D; (**B**) CPMG; (**C**) Diffusion.

Using PCA-CA-kNN supervised analysis, the metabolomic profiles of the relapse-free patients in the eCRC cohort (*n* = 65) and the patients in the mCRC cohort (*n* = 75) were classified. The model showed significant differential clustering, with good separation of the two groups (Figure 2). Clustering was achieved by the CPMG, NOESY1D, and Diffusion spectra. The clustering provided by the NOESY spectra showed the highest accuracy in the discrimination, with 70.0% accuracy, 70.7% sensitivity, and 69.2% specificity, compared with 67.8%, 65.3%, and 70.8% for CPMG and 62.8%, 64.0%, and 61.5% for Diffusion-edited, respectively.

### 3.3. Metabolomics Analysis of Relapsed Patients in the eCRC Cohort

**Figure 3:**
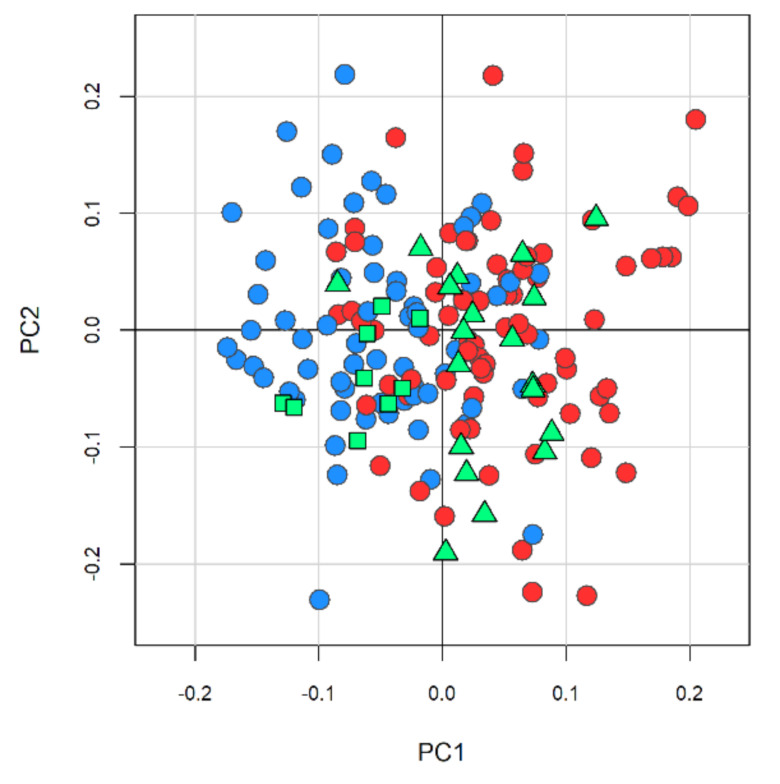
Prediction of cancer relapse using the NMR metabolomic fingerprint. The model calculated on NOESY1D spectra, discriminating patients free from eCRC disease relapse (light blue, dots, *n* = 65) and patients with mCRC (red dots, *n* = 75) using the PCA-CA-kNN analysis, was used to classify patients who developed disease recurrence. Twenty (green triangles) out of 29 patients were correctly classified as “metastatic” disease, the other nine patients (green squares) were mis-classified as early disease. This model provides an overall prediction accuracy of 69%.

Using the NOESY1D model, we analyzed the remaining patients in the eCRC cohort (*n* = 29) who experienced disease relapse, with the hypothesis that their metabolomic fingerprint would more closely resemble that of patients with mCRC. Using this model, 20 out of 29 patients with relapse clustered in the PCA-CA-kNN subspace of the metastatic group, meaning that 69.0% of the patients with relapse in the eCRC cohort were predicted as “metastatic” based on the resemblance with the profiles of the metastatic samples. The remaining nine patients with disease relapse were mis-classified as eCRC free from relapse (Figure 3).

### 3.4. Univariate Metabolite Analysis 

**Figure 4:**
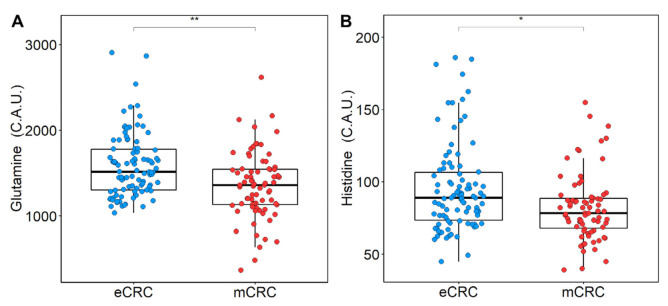
Boxplots of glutamine (**A**) and histidine (**B**) levels in patients with eCRC (blue) and mCRC (red), *p*-values were obtained using Wilcoxon test and adjusted for FDR. * *p* < 0.05, ** *p* < 0.01.

Thirty-two metabolites were quantified in all serum spectra. The differences in concentrations between patients with eCRC and mCRC are reported in Table 2. Significantly lower levels of glutamine and histidine were observed in patients with mCRC (Figure 4). The individual discrimination power of glutamine and histidine was tested by setting the threshold at their medians: 60.7% and 58.7% discrimination accuracies were obtained, respectively.

### 3.5. Prognostic Significance of the Metabolomic Classifier in the eCRC Cohort

We next assessed the prognostic role of the metabolomic classifier by using the metabolomics classification of the whole eCRC cohort. Patients were therefore classified as having a “high” or “low” metabolomic risk score according to each individual metabolomic profile being more or less close to the mCRC metabolomic space, respectively. Forty patients with eCRC were classified as “high risk” by their metabolomic fingerprint, with 50% of patients eventually developing disease relapse in this group, versus only nine out of 45 patient who relapsed (17%) in the “low risk” metabolomic group. In terms of clinicopathological features, in the metabolomic “high risk” group, half had stage III disease whereas in the low metabolomic risk group, the majority of patients had stage II (52%). The three patients with stage I disease were all classified at low metabolomic risk. In accordance with the distribution of tumor stage between high/low metabolomic risk, adjuvant chemotherapy was received by a higher proportion of patients (80%) with high metabolomic risk, compared with 59% of patients with low metabolomic risk (Table 3). Of note, six out of the eight patients with eCRC treated with neo-adjuvant chemo-radiotherapy were classified at high metabolomic risk, and two of these developed cancer recurrence.

**Figure 5:**
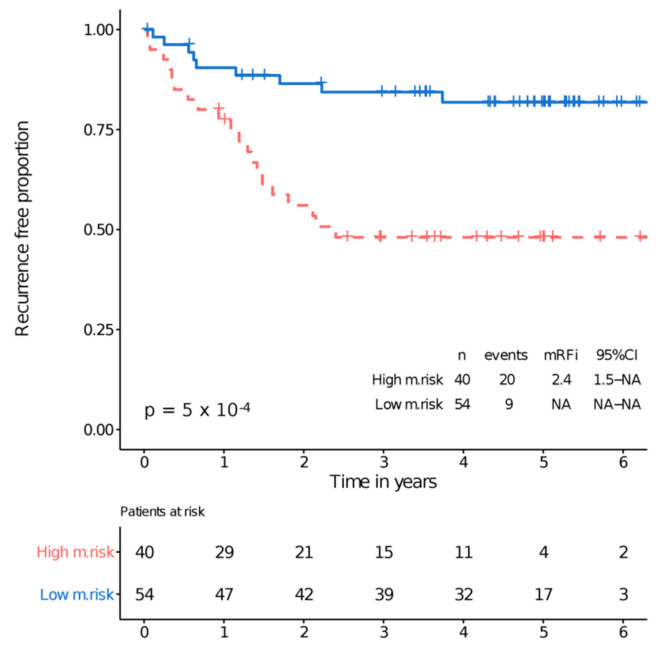
RFI by metabolomic risk.

RFI was significantly different between high and low metabolomic risk (*p* = 5 × 10^−4^) (Figure 5). Similar results were obtained for DFS (Figure S2) and CRC specific survival (Figure S3).

**Figure 6:**
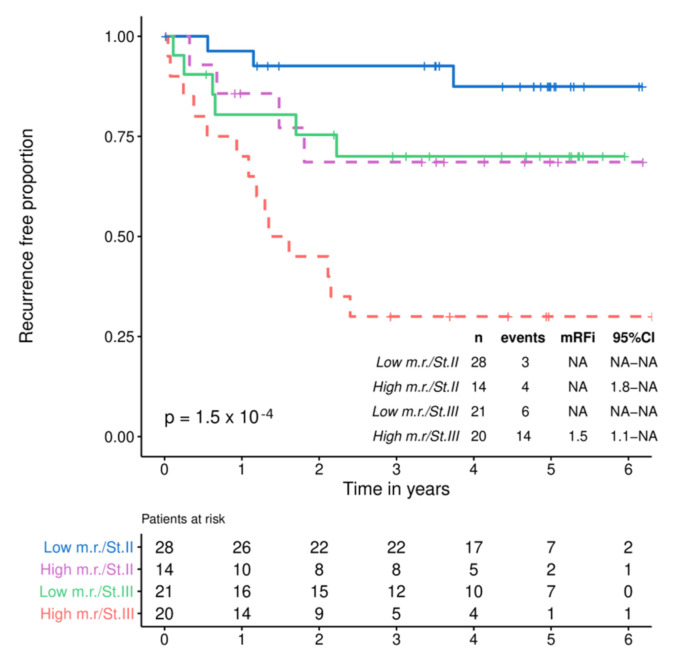
RFI by metabolomic risk in stage II and III.

Interestingly, similar outcomes were observed in patients with pathological stage II disease plus high metabolomic risk and patients with pathological stage III disease plus low metabolomic risk (Figure 6).

The prognostic effect of the metabolomic classifier was independent of the stage of disease as shown by the multivariate analysis (Table 4).

## 4. Discussion

Currently, management of early stage colorectal cancer relies upon TNM staging as the most informative instrument for the prediction of the risk of relapse and potential benefit derived from adjuvant treatment [60,61,62,63,64,65,66]. In the era of precision medicine, and despite the availability of more detailed molecular information derived from the analysis of primary tumors, there is an ongoing need to define new risk-stratification tools to improve prognostication for patients with eCRC.

Over the last decade, there has been increasing attention on the use of liquid biopsy as a non-invasive means of obtaining new potential prognostic and predictive information in early and advanced colorectal cancer. Analysis of ctDNA identifies cancer-specific mutations, and is emerging as a promising tool for predicting relapse, monitoring response to treatment, and tracking resistance to anti-EGFR-based therapy in the metastatic setting [67].

Serum cancer metabolomics is a powerful tool to reliably obtain a complex molecular representation of the tumor phenotype, which accounts both for tumor cell-derived and host-derived signals, such as those emerging from tumor micro-environment, inflammatory and immune responses [41]. Via the analytical approach of gas chromatography time-of-flight mass spectrometry (GC–TOF-MS)-based metabolomics, Qiu et al identified a panel of 15 altered metabolites in a large cohort of patients with colorectal cancer, which was able to predict the rate of relapse and survival for patients after surgery and chemotherapy [44].

In this analysis, we present a retrospective evaluation of a well-characterized cohort of elderly patients with colorectal cancer treated at our institution and with a long-term follow up with the aim of exploring the role of NMR serum metabolomics fingerprinting in predicting relapse. We pooled samples of elderly patients with CRC included in three clinical onco-geriatric trials that incorporated the collection of serum samples for metabolomics analysis.

In this study, using the PCA-CA-kNN supervised analysis, the serum metabolomic fingerprint of 65 patients with eCRC free from disease relapse for at least 3 years and 75 mCRC patients were classified, showing significant differential clustering with good separation of the two groups, with higher accuracy using the NOESY spectra (70.0% accuracy, 70.7% sensitivity, and 69.2% specificity). Interestingly, both low molecular weight metabolites and high molecular weight lipoproteins and proteins contributed to the discrimination between the two groups of patients, suggesting that an approach considering both these components may be more informative as compared to other more selective approaches. 

Histidine and glutamine were shown to be significantly decreased in the serum samples from patients with metastatic disease. Glutamine plays an anaplerotic role by replenishing TCA cycle intermediates, and thus it serves as an opportunistic fuel source for cancer cells [68,69]. In particular, we observed a decreasing trend of glutamine levels from samples from patients free from disease, to those with relapsed eCRC, and to mCRC. This observation suggests an association between glutamine and cancer progression, corroborating previous evidence that low glutamine levels are associated with advanced cancer stage and with poor cancer-specific survival [70]. Downregulation of histidine has been observed in previous studies [71,72], and it can be induced by the acceleration of decarboxylation from histidine to histamine, since in CRC, histidine decarboxylase shows increased activity [73]. Histamine can act as an angiogenic factor, and it also seems to exert an immunosuppressive effect on the local response against cancer [74].

It is worth noting that none of the aforementioned metabolites had sufficient independent diagnostic power, as demonstrated by their accuracies in the discrimination between samples from patients free from disease recurrence and those with mCRC. However, both are important contributors in the metabolic fingerprint of patients with CRC, and this is an innovative point of the approach described here. The metabolic fingerprint can be thought as a sort of holistic super-biomarker with a discriminative power higher than the simple sum of the few quantified metabolites, as it takes into account all the detectable signals of metabolites/lipoproteins present in the NMR spectra [75].

Based on the resemblance with the metastatic fingerprint, our model was able to correctly classify at high risk of disease recurrence 69% of the patients with early stage disease who eventually developed disease recurrence. This suggests that the metabolomic fingerprint of patients with eCRC who will experience cancer recurrence presents with a sort of prospective “metastatic signature”, even in the absence of clinically evident metastatic spread. Furthermore, analyzing the metabolomic classification of all patients with eCRC (both free from disease and relapsed) with Kaplan–Meier curves, a strong prognostic effect was observed, with patients who displayed a metabolomic profile that was closer to that of patients with metastatic disease (i.e., a “high risk” metabolomic score) having a significantly higher probability of disease relapse as compared to those with a low risk metabolomic score. It is notable that the group of patients with a high-risk metabolomic score was rich in patients with known adverse clinicopathological prognostic factors (higher disease stage, greater nodal involvement) as compared to the group with a low risk metabolomic profile. However, the prognostic effect of the metabolomic fingerprint was independent of stage, as shown by the multivariate analysis and the subgroup analysis by stage. Although we could not fully assess all the clinicopathological characteristics that may define conventional risk stratification in patients with stage II disease (e.g., presence of perineural and/or vascular invasion, number of examined lymph nodes, presence of bowel obstruction at disease onset), patients with stage II disease plus a low-risk metabolomic profile had an excellent outcome. Interestingly, the prognosis of patients with stage II disease plus a high-risk metabolomic score was comparable to that of patients with stage III disease plus a low-risk metabolomic score. These data support the potential usefulness of metabolomics for identifying a more precise risk profile in patients with eCRC.

Metabolomic analysis by NMR enables rapid and reproducible characterization of the eCRC serum metabolic fingerprint associated with a high risk of cancer relapse, thus refining risk stratification to that achieved by considering classical TNM staging alone, and potentially identifying those patients who will benefit most from adjuvant treatment. As such, NMR metabolomics could represent a valid addition to established risk stratification instruments used in current practice.

In this analysis, we used a similar approach to that employed in previously published analyses in the context of early breast cancer [49,50,51]. These data, together with those from other groups [42,43,44,45,46], collectively provide evidence that serum metabolomics may be a valuable tool for prognosis determination across multiple cancer types. Although this current study provides important information in the setting of eCRC prognostic evaluation, there are certain limitations. Firstly, the analysis had a retrospective nature, with a small number of patients and relatively few events. Secondly, in many cases, only partial information about clinicopathological features was available. The group of patients with eCRC free from disease at follow up included six patients treated with neo-adjuvant chemotherapy. Therefore, the serum samples from these patients were collected before the commencement of neoadjuvant chemotherapy but whilst the tumor was still in situ. The presence of the tumor could produce noise in the metabolic fingerprint of the CRC cohort, in turn decreasing the potential of NMR-based metabolomics to detect micro-metastatic disease. However, due to the limited sample size of the present study, we opted to retain these samples in our analyses, accepting the possibility that results may be slightly underestimated.

It is worth of noting that this case series analyzes an elderly population in which the joint assessment of risk factors and general health conditions of each individual has a meaningful impact on therapeutic decision-making and on the choice of adjuvant therapy regimen. This aspect limits the extension of our findings to a general population not selected based on age, but it reflects a real-life situation and thus, our data show how NMR metabolomics can be effectively applied in the clinical practice.

In order to validate these findings in a general population, we are conducting a prospective trial in a larger and multicentric population, focused on high-risk stage disease, the LIBIMET (LIquid BIopsy and METabolomics in colon cancer) study (ethics approval 11252_BIO). The primary endpoint of LIBIMET is to redefine the risk of relapse in patients with early stage colon cancer by employing serum metabolomic profiles and ctDNA.

## 5. Conclusions

NMR metabolomic fingerprinting can discriminate between early and metastatic CRC in elderly patients and may represent a useful tool to build a prognostic model capable of assessing the likelihood of disease relapse, based on the degree to which a serum profile derived from a patient with early disease resembles that of a metastatic profile. Based on these results, we have opened a prospective trial, with the aim of testing the potential value of metabolomic profiling in predicting disease relapse in a larger population of patients with high-risk stage II and stage III colorectal cancer.

## Figures and Tables

**Table 1:** Baseline characteristics of the eCRC population.

Covariate	Study Patients (*N* = 94)
Age at study entry, years	
Median age (range)	78 (70–89)
Tumor localization; *n* (%):	
• Left/Rectum	57 (61)
• Right	37 (39)
Tumor size; *n* (%):	
• T1	1 (1)
• T2	6 (6)
• T3	72 (77)
• T4	7 (7)
• NA *	8 (9)
Nodal involvement; *n* (%):	
• N0	41 (44)
• N1	28 (30)
• N2	17 (18)
• NA *	8 (9)
Histologic grade; *n* (%):	
• Grade 1	6 (6)
• Grade 2	67 (72)
• Grade 3	17 (18)
• NA	4 (4)
Stage; *n* (%):	
• Stage I	3 (3)
• Stage II	42 (45)
• Stage III	41 (43)
• NA *	8 (9)
Relapse; *n* (%):	
• Not relapsed	65 (69)
• Relapsed	29 (31)
Treatment; *n* (%):	
• Neoadjuvant chemo-radiotherapy	8 (9)
• Adjuvant CT	56 (59)
• No treatment	30 (32)

med [Q1; Q3] (min; max) for numerical variables *N* (%) for categorical variables. * patients with rectal cancer pre-treated with neoadjuvant chemo-radiotherapy.

**Table 2:** Metabolite univariate analysis for the comparison between eCRC and mCRC patients.

Metabolite	*p*-Value	*p*-Value FDR Adjusted	Effect Size
Glutamine	0.0002	0.007	0.330
Histidine	0.002	0.028	0.280
Formate	0.018	0.194	−0.212
Alanine	0.061	0.320	0.168
Proline	0.062	0.320	0.168
Valine	0.069	0.320	0.163
3-methyl-2-oxovalerate	0.070	0.320	−0.163
Tyrosine	0.122	0.451	0.139
Acetate	0.127	0.451	−0.137
Glucose	0.161	0.514	0.126
Isoleucine	0.193	0.561	0.117
3-hydroxybutyrate	0.234	0.619	−0.107
Leucine	0.251	0.619	0.103
Glycoproteins	0.281	0.637	0.097
Lactate	0.299	0.637	−0.093
Lipoproteins βCH_2_	0.403	0.752	0.075
Lipoproteins N(CH_3_)_3_	0.417	0.752	0.073
cholesterol	0.434	0.752	0.070
Creatinine	0.447	0.752	0.068
Citrate	0.472	0.755	0.065
Lipoproteins CHCH	0.572	0.833	0.051
Glutamate	0.614	0.833	−0.045
Lipoproteins CH_2_n	0.618	0.833	0.045
N,N-Dimethylglycine	0.625	0.833	−0.044
Lipoproteins CHCH_2_CH	0.661	0.846	0.039
Lipoproteins CH_3_	0.698	0.859	0.035
Pyruvate	0.760	0.901	−0.028
Phenylalanine	0.792	0.905	−0.024
Dimethylsulfone	0.846	0.911	−0.018
Glycine	0.896	0.911	−0.012
Lipoproteins CHCH_2_CH_2_	0.898	0.911	0.012
Creatine	0.911	0.911	−0.010

*p*-value, *p*-value adjusted for multiple comparisons, and Cliff’s Delta effect size are reported in table for each metabolite. An adjusted *p*-value < 0.05 is considered statistically significant. Positive effect size means lower levels in mCRC serum samples.

**Table 3:** Patient and disease characteristics by metabolomic risk (eCRC cohort).

Covariate	Whole Sample (*N* = 94)	High (*N* = 40)	Low (*N* = 54)	*p*-Value
Age at study entry (years)	78 (70–89)	76 (70;85)	78 (71;89)	0.062
Tumor localization (*n*,%)				0.025
• Left/Rectum	57 (61)	19 (48)	38 (70)	
• Right	37 (39)	21 (52)	16 (30)	
Tumor size (*n*,%)				0.273
• T1	1 (1)	0 (0)	1 (1)	
• T2	6 (6)	3 (8)	3 (6)	
• T3	72 (77)	29 (72)	43 (80)	
• T4	7 (7)	2 (5)	5 (9)	
• NA *	8 (9)	6 (15)	2 (4)	
Nodal involvement (*n*,%)				0.017
• N0	41 (44)	11(28)	30 (56)	
• N1	28 (30)	16 (40)	12 (22)	
• N2	17 (18)	7 (18)	10 (19)	
• NA *	8 (9)	6 (15)	2 (4)	
Histologic grade (*n*,%)				0.495
• Grade 1	6 (7)	1 (3)	5 (10)	
• Grade 2	67 (74)	30 (79)	37 (71)	
• Grade 3	17 (19)	7 (18)	10 (19)	
• NA	4	2	2	
Stage (*n*,%)				0.054
• Stage I	3 (2)	0 (0)	3 (6)	
• Stage II	42 (45)	14 (35)	28 (51)	
• Stage III	41 (44)	20 (50)	21 (39)	
• NA *	8 (9)	6 (15)	2 (4)	
Neo/adjuvant CT (*n*,%)				0.033
• Yes	64 (68)	32 (80)	32 (59)	
• No	30 (32)	8 (20)	22 (41)	
Relapse (*n*,%)				0.001
• Not relapsed	65 (69)	20 (50)	45 (83)	
• Relapsed	29 (31)	20 (50)	9 (17)	

med [Q1; Q3] (min; max) for numerical variables *N* (%) for categorical variables. * patients treated with neoadjuvant chemo-radiotherapy

**Table 4:** Multivariate analysis.

	UNIVARIATE (*n* = 84)			MULTIVARIATE (*n* = 84)		
	HR	95% CI	*p*-Value	HR	95% CI	*p*-Value
Metabolomic risk (High vs. Low)	3.68	1.65–8.22	0.001	3.18	1.41–7.15	0.005
Stage (III vs. I–II)	3.57	1.51–8.46	0.004	3.05	1.28–7.28	0.012
Histologic grade (G3 vs. G1–G2)	1.71	0.72–4.04	0.2			
Cancer localization (Right vs. Left/Rectum)	0.97	0.45–2.08	> 0.9			
Adjuvant chemotherapy (No vs. Yes)	0.53	0.21–1.31	0.2			
Tumor dimension (T3–T4 vs. T1–T2)	2.34	0.32–17.3	0.4			

HR = Hazard Ratio, CI = confidence interval criteria for entry and stay in the multivariate model is *p* < 0.05. *N* patients with complete data for selected covariates *n* = 84.

## Data Availability

The data presented in this study are available on reasonable request from the corresponding author.

## Supplementary Materials

The following are available online at https://www.mdpi.com/article/10.3390/cancers13112762/s1, Figure S1: A) RFI by stage; B) DFS by stage. Figure S2: DFS by metabolomic risk. Figure S3: A) CRC specific survival by stage B) CRC specific survival by metabolomic risk
