# Supplementary material for: A Serum Metabolomics Classifier Derived from Elderly Patients with Metastatic Colorectal Cancer Predicts Relapse in the Adjuvant Setting
Source: Cancers (Basel). 2021 Jun 2;13(11):2762. doi: 10.3390/cancers13112762 (PMC8199587; doi:10.3390/cancers13112762)
Supplement: Supplementary file 1 [file cancers-13-02762-s001.zip › cancers-1204565-supplementary.pdf]

cancers MDPI

Supplementary Materials: A Serum Metabolomics Classifier Derived From Elderly Patients with Metastatic Colorectal Cancer Predicts Relapse In the Adjuvant Setting

Samantha Di Donato, Alessia Vignoli, Chiara Biagioni, Luca Malorni, Elena Mori, Leonardo Tenori, Vanessa Calamai, Annamaria Parnofiello, Giulia Di Pierro, Ilenia Migliaccio, Stefano Cantafio, Maddalena Baraghini, Giuseppe Mottino, Dimitri Becheri, Francesca Del Monte, Elisangela Miceli, Amelia McCartney, Angelo Di Leo, Claudio Luchinat and Laura Biganzoli

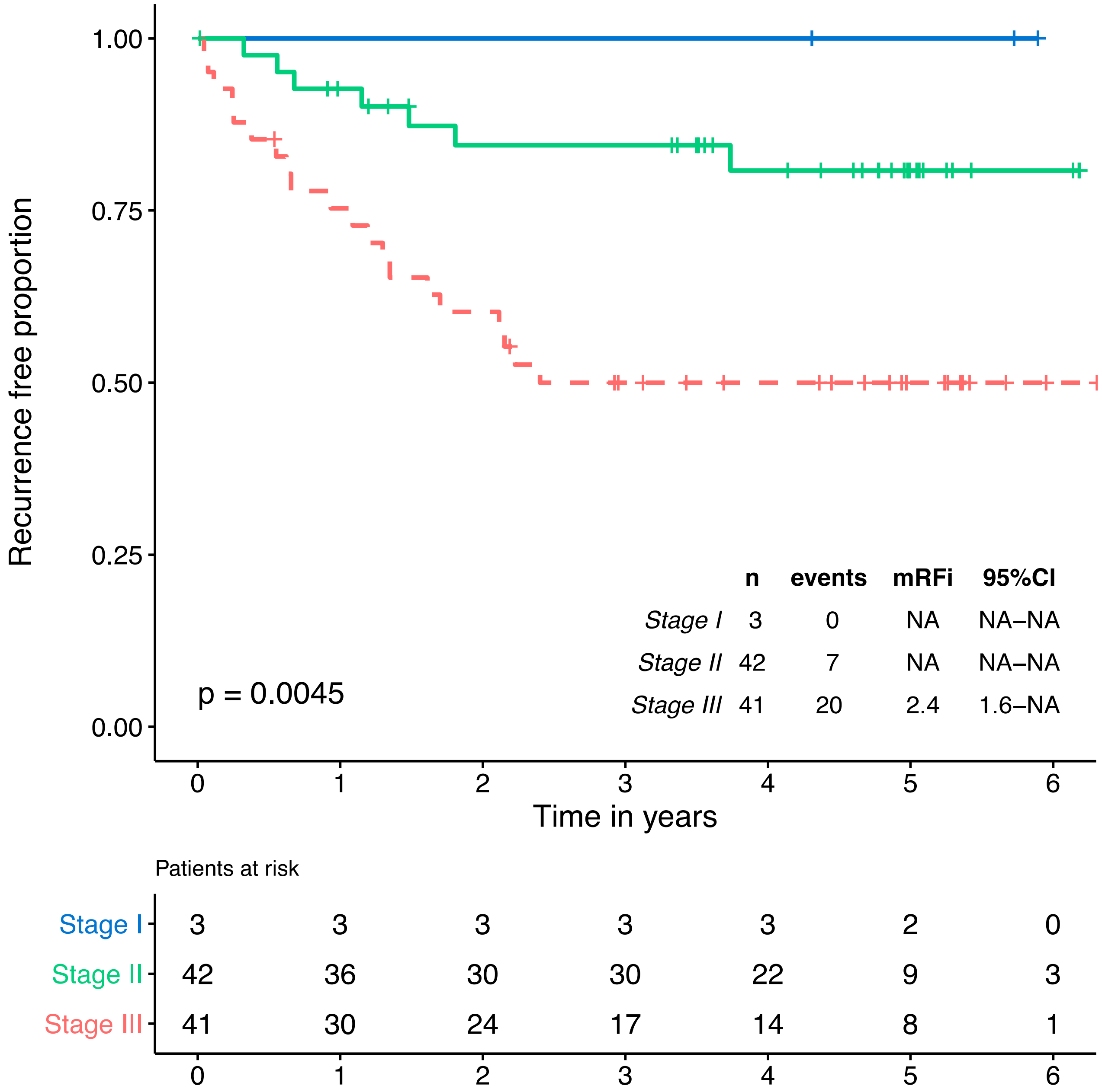

Figure S1. A-B (A) RFI by stage.

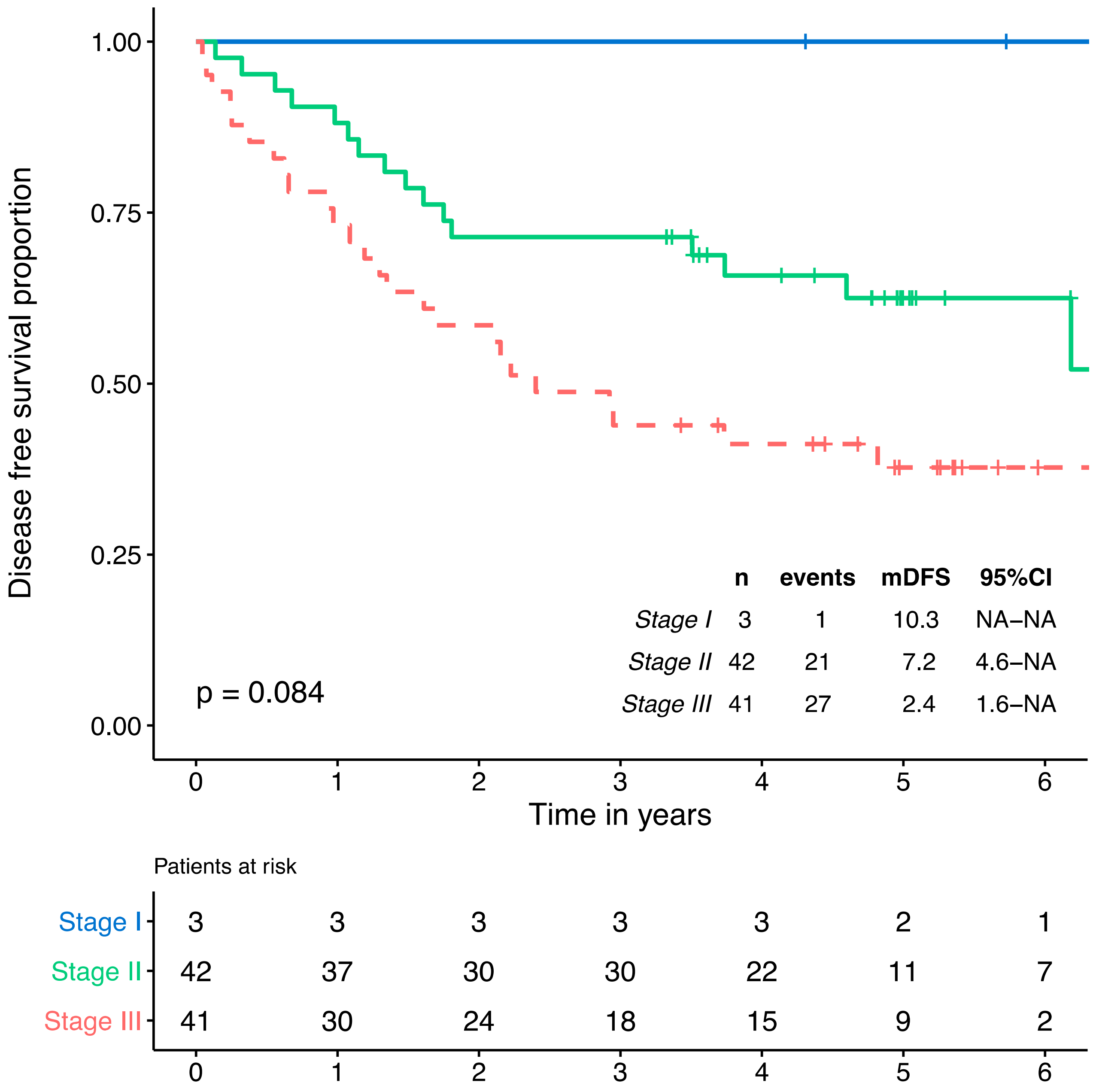

(**B**) DFS by stage.

Figure S1.

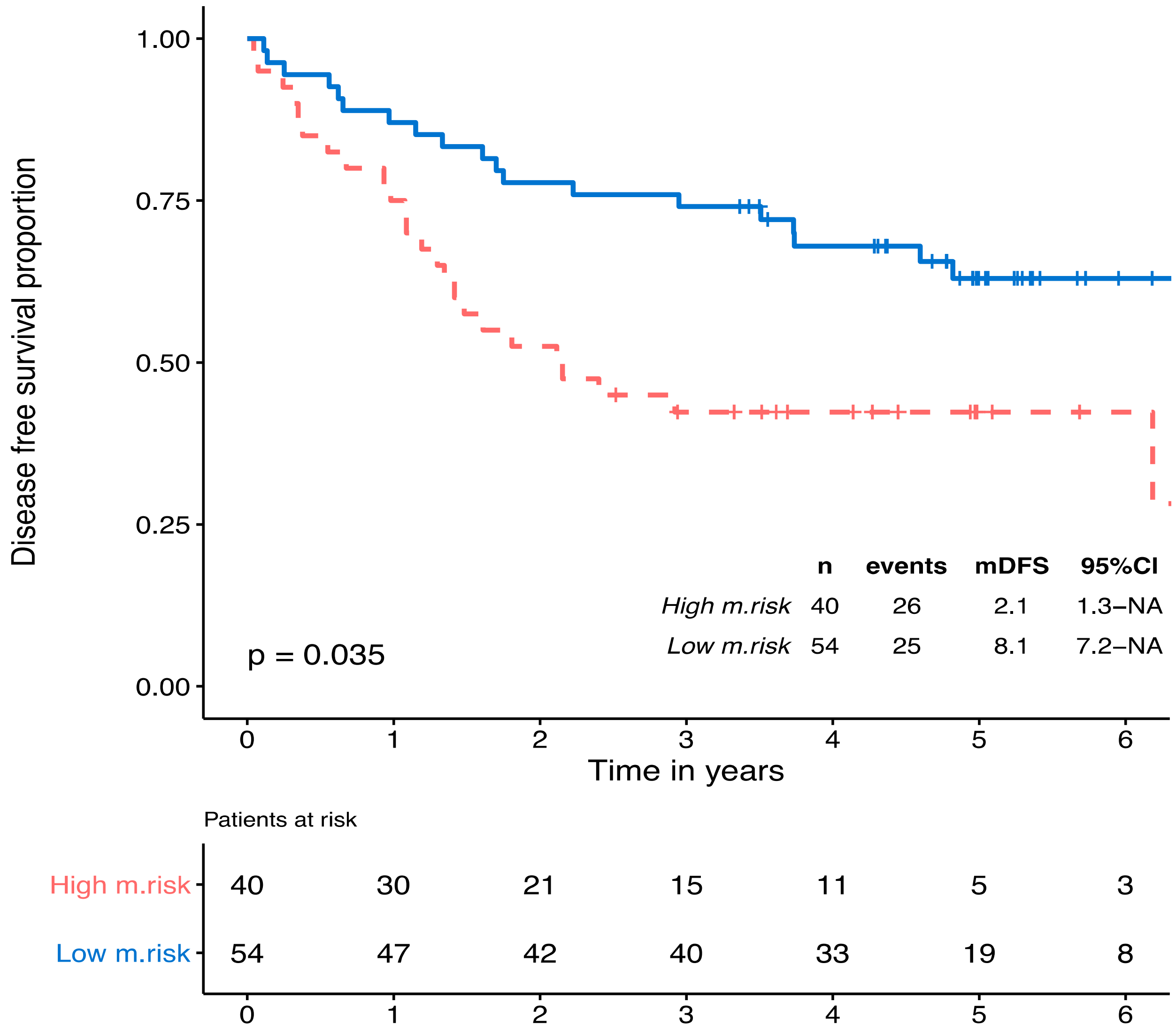

Figure S2. DFS by metabolomic risk.

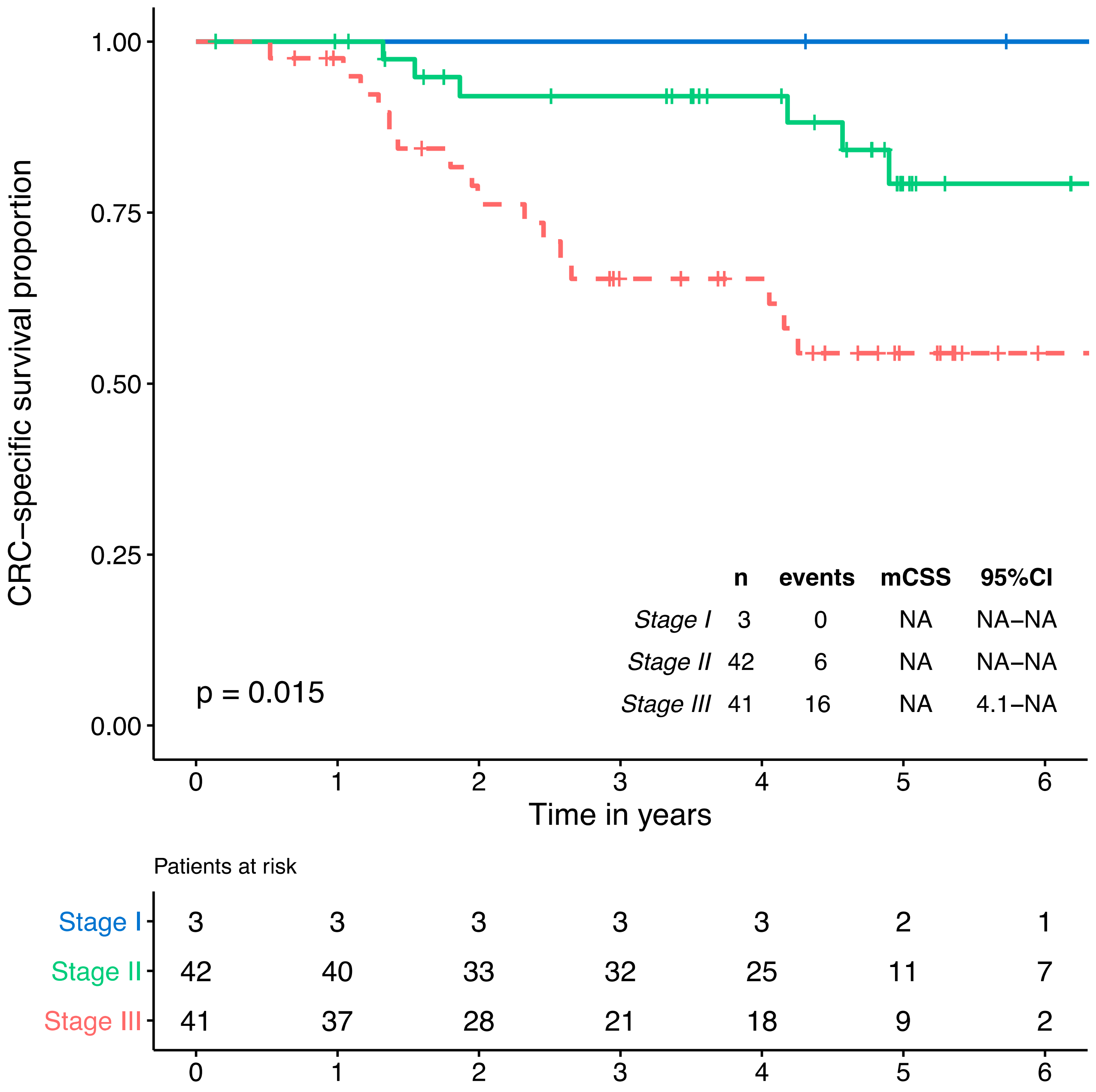

Figure S3.A -B (**A**) CRC specific survival by stage.

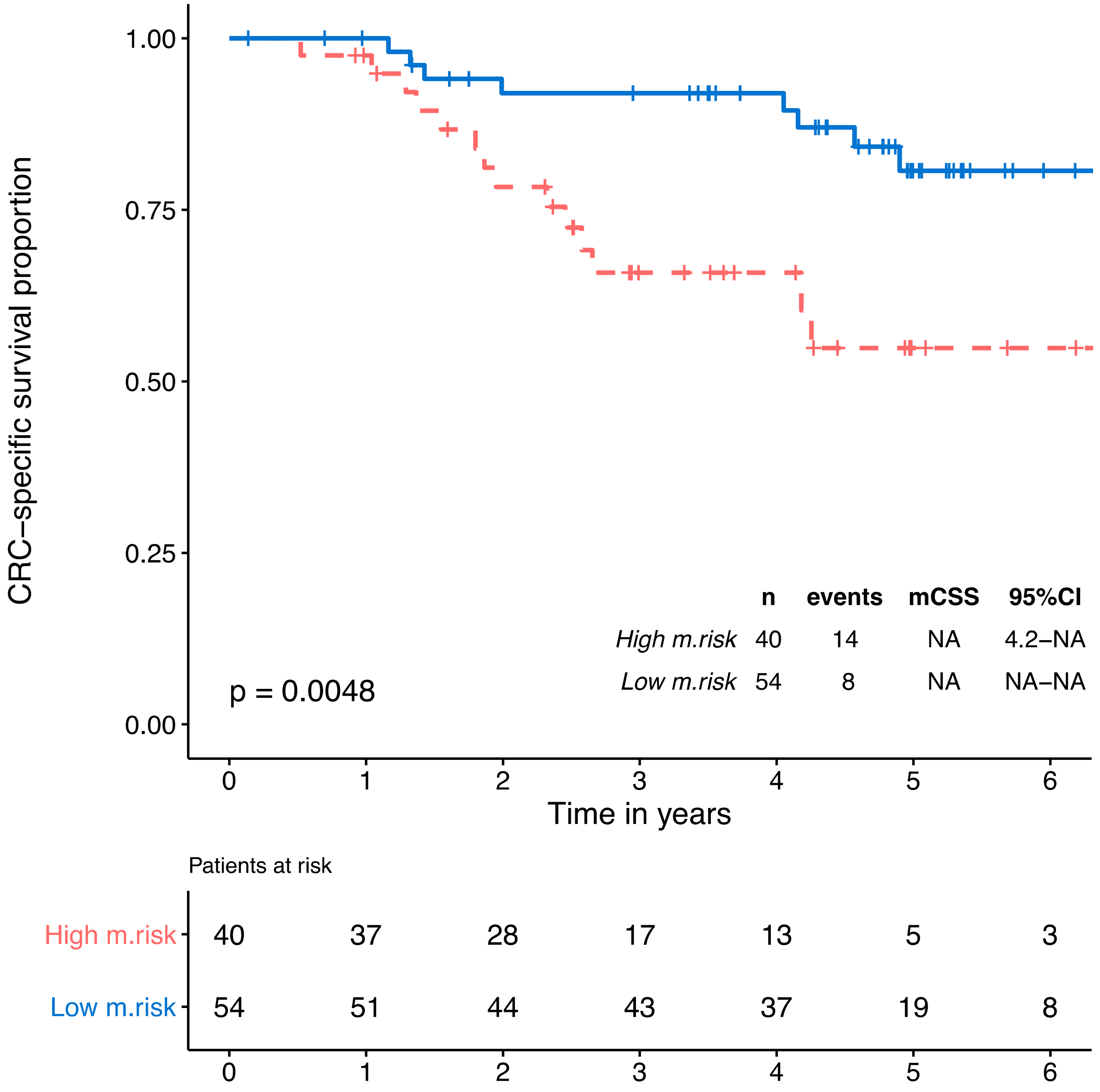

(**B**) CRC specific survival by metabolomic risk.

Figure S3.

Table S1. Metastatic cohort: descriptive table by primary site.

		Left/rectum	Right	Unknown	Whole sample
		(*N* =36)	(*N* = 37)	(*N* = 2)	(*N* = 75)
Age at study entry	Median (min;max)	77 (70;86)	78 (70;86)	79 (75;84)	77 (70;86)
Gender					
	Female	13 (36%)	20 (54%)	0 (0%)	33 (44%)
	Male	23 (64%)	17 (46%)	2 (100%)	42 (56%)
BRAF mutational status					
	Mutated	1 (4%)	2 (10%)	0 (0%)	3 (7%)
	Wild type	24 (96%)	19 (90%)	0 (0%)	43 (93%)

	Missing	11	16	2	29
KRAS mutational status					
	Mutated	16 (64%)	11 (52%)	0 (0%)	27 (59%)
	Wild type	9 (36%)	10 (48%)	0 (0%)	19 (41%)
	Missing	11	16	2	29
NRAS mutational status					
	Mutated	2 (11%)	1 (6%)	0 (0%)	3 (9%)
	Wild type	17 (89%)	15 (94%)	0 (0%)	32 (91%)
	Missing	17	21	2	40
Line of metastatic treatment					
	0	4 (11%)	5 (14%)	0 (0%)	9 (12%)
	1	30 (83%)	27 (73%)	1 (50%)	58 (77%)
	2	2 (6%)	3 (8%)	1 (50%)	6 (8%)
	3	0 (0%)	2 (5%)	0 (0%)	2 (3%)
